# Biomechanical differences in lower limb movements during lifting tasks before and after fatigue

**DOI:** 10.3389/fbioe.2025.1702269

**Published:** 2025-10-29

**Authors:** Sen Yang, Yingjian Zhang, Jie Cai

**Affiliations:** ^1^ Capital University of Physical Education and Sports, Beijing, China; ^2^ Langfang Normal University, Langfang, Hebei, China

**Keywords:** lifting task, fatigue, kinematics, kinetics, movement patterns

## Abstract

**Objective:**

To investigate the effects of fatigue on lower limb kinematics and kinetics during manual lifting tasks and to quantitatively analyze these effects in order to provide guidance for safe work practices.

**Methods:**

Twenty healthy male college students performed lifting tasks with two load conditions (15 kg, low load; and 25 kg, high load) before and after fatigue. An eight-camera 3D motion capture system and two force plates were used to collect surface marker trajectories and ground reaction force data. Inverse kinematics and inverse dynamics analyses were conducted using OpenSim to calculate movement duration, joint angles, joint angular velocities, joint moments, joint power, and joint energy expenditure.

**Results:**

(1) For the 15 kg lifting task, there were no significant differences in any parameter between pre- and post-fatigue conditions. (2) For the 25 kg task, compared to the pre-fatigue state, subjects exhibited decreased movement duration, increased joint range of motion, faster angular velocities, and elevated joint power and energy expenditure after fatigue.

**Conclusion:**

Under low load conditions, the primary kinematic and kinetic parameters of the lower limb joints remained stable before and after fatigue, demonstrating strong fatigue resistance. In contrast, under high-load conditions, fatigue altered the lower limb movement patterns. The combined effect of high load and fatigue not only increased the burden on the musculoskeletal system but also led to a rise in potential injury risk, which requires further research for validation.

## Introduction

In labor-intensive industries (e.g., construction, agriculture, logistics), manual material handling (hereafter referred to as lifting tasks) is a common physical labor form, yet it poses high risks of work-related musculoskeletal disorders (WMSDs) due to inappropriate postures and fatigue (Portell et al., 2019; Sauter et al., 2021). Notably, WMSD prevalence reaches 68% among construction workers (Schneider, 2001), and 49.9% among manufacturing workers (Da Costa et al., 2015), with lifting also being a key contributor to low back pain (Kuiper et al., 1999). As the primary force-transmitting and posture-stabilizing structures during lifting, lower limbs’ biomechanical abnormalities are closely linked to such injuries—making it critical to clarify how fatigue alters lower limb movement patterns in lifting tasks for worker health protection.

The U.S. National Institute for Occupational Safety and Health identifies biomechanical factors as the most important cause of lifting-related musculoskeletal injuries (Waters et al., 1993). Biomechanical analysis of lifting can reveal lower limb loads (e.g., joint moments, ground reaction forces), identify risk factors (e.g., excessive knee/hip range of motion), and inform optimization measures (Marras et al., 2000). Evaluating joint motion, force transmission, and postural stability during lifting further enables prediction and prevention of injuries from improper operations (Antwi-Afari et al., 2017).

Muscle fatigue, or perceived fatigue, is a natural outcome of physical activity and exercise that can lead to decreased physical function (Enoka and Duchateau, 2016). During prolonged lifting operations—characterized by repetitive bending, extending, and load-bearing of the lower limbs—workers are prone to fatigue due to continuous activation of lower limb muscles (e.g., quadriceps, hamstrings). Similar to long-term standing, such prolonged load-bearing tasks can induce not only lower limb fatigue but also discomfort in the feet, calves, and lower back, with these symptoms worsening over time and potentially exacerbating injury risk (Lu et al., 2021). Fatigue not only reduces operational efficiency, but—more importantly—it has been clearly identified as an independent risk factor for lifting-related injuries (Sauter et al., 2021). Specifically, fatigue-induced declines in lower limb muscle strength and joint proprioception may disrupt the coordination between hip, knee, and ankle joints during lifting, leading to altered movement rhythms or excessive joint loading (Sparto et al., 1997). Notably, this biomechanical disruption is analogous to the trunk extensor fatigue-induced impairment of spinal stability observed in previous studies, where fatigue reduced the neuromuscular system’s ability to respond to local perturbations, increasing the likelihood of abnormal intervertebral motion (Larson et al., 2018). Therefore, developing strategies to identify and prevent the harmful consequences of fatigue in lifting tasks is essential (Dupuis et al., 2024). Studies have shown that accumulated fatigue affects both physical and cognitive functions, leading to changes in muscle function as well as the central nervous system’s ability to plan voluntary movements, ultimately resulting in altered motor control (Enoka and Duchateau, 2016; Dupuis et al., 2022). For instance, in lifting-like repetitive lower limb movements, fatigue has been associated with increased hip flexion angles and delayed knee extension timing—changes that may elevate injury risk (Thomas et al., 2010). While studies in running (e.g., decreased step frequency with fatigue) provide general insights into fatigue-related motor changes (Winter et al., 2016), the unique load-bearing and multi-joint coordination demands of lifting tasks require targeted investigation of lower limb biomechanics.

In the field of exercise science, fatigue-induced changes in motor control and their effects on the mechanical loading of the musculoskeletal system (e.g., tendons, muscles, cartilage) are well recognized (Hodges and Tucker, 2011). Fatigue has been shown to reduce muscle strength and joint proprioception, increase joint laxity, and consequently weaken joint stability—effects that may be amplified in lifting tasks due to the additional external load. (Lu et al., 2024; Zhu et al., 2024). However, there is still considerable academic debate regarding whether these changes are ultimately positive or negative, and this debate extends to lifting-specific scenarios. For example, in studies of anterior cruciate ligament (ACL) injury, the effects of fatigue have yielded diverse conclusions. Some studies have found that fatigue significantly alters knee joint mechanics, increasing the risk of ACL injury (Collins et al., 2016; Borotikar et al., 2008), a risk associated with reduced muscle coordination, lower efficiency of force transmission, and diminished knee control caused by fatigue. Conversely, other studies have pointed out that individuals may actively adjust their movement patterns or adopt protective strategies (such as reducing high-load movements or limiting knee joint range of motion) under fatigue, in order to decrease joint loading and lower the risk of injury (Cortes et al., 2014; Rolley et al., 2023). Extrapolating to lifting tasks, similar uncertainties exist: it remains unclear whether fatigue leads to harmful changes (e.g., excessive hip/knee moments) or adaptive protective adjustments in lower limb kinematics/kinetics. Additionally, some research has suggested that fatigue’s effects may be influenced by individual movement strategies, physical fitness, and training levels (Barber-Westin and Noyes, 2017)—factors that further complicate the understanding of fatigue-lifting biomechanics interactions.

Several biomechanical studies on lifting tasks have compared the biomechanical differences associated with varying loads and postures (Marican et al., 2025; Xiang et al., 2023). However, research on fatigue and injury in this context has mainly adopted questionnaire-based or prospective cohort designs (Bláfoss et al., 2023; Mayer et al., 2024). These methods have considerable limitations: questionnaire-based studies rely on subjective self-reports of fatigue and injury, which are prone to recall bias and cannot quantify real-time biomechanical changes during lifting; prospective cohort studies can establish injury-fatigue associations but fail to identify the underlying biomechanical mechanisms (e.g., how fatigue alters joint moments to cause injury). In contrast, experimental studies using 3D motion capture and force plates can directly measure lower limb kinematic (e.g., joint angles) and kinetic (e.g., joint power) parameters during lifting, enabling objective analysis of fatigue’s biomechanical effects—thus addressing the gaps of previous methods.

Based on the above research gaps, this study proposes the following hypotheses regarding lower limb biomechanics during lifting tasks: (1) Under low-load lifting conditions, fatigue will not significantly alter key lower limb kinematic/kinetic parameters due to the relatively low muscle demand; (2) Under high-load lifting conditions, fatigue will lead to significant changes in lower limb biomechanics, thereby increasing musculoskeletal load. The purpose of this study is to test these hypotheses by investigating the effects of fatigue on the lower limb kinematics and kinetics during lifting tasks and to quantitatively analyze these effects. While this study uses healthy university students as subjects (with limitations in generalizing to industrial workers), the findings may still provide a foundational understanding of fatigue-lifting biomechanical interactions, supporting the development of targeted motor control strategies for workers and reducing the incidence of musculoskeletal injuries.

## Methods

### Participants

Twenty healthy male participants were recruited for this study, all of whom were students from Capital University of Physical Education and Sports. The average age of the participants was 19.05 ± 1.15 years, with a mean body weight of 69.80 ± 4.93 kg and a mean height of 172.15 ± 2.96 cm. All participants had no history of lower limb injuries. To avoid pre-experimental fatigue, no intense physical activity was performed before testing. None of the subjects had previous experience with similar experiments. All participants provided written informed consent prior to participation. The protocol was approved by the Ethics Committee of Capital University of Physical Education and Sports (2024A059).

## Data collection and experimental procedure

A three-dimensional motion capture system (OptiTrack, 8-camera, NaturalPoint, United States) was used to collect the spatial coordinates of anatomical landmarks at a sampling rate of 200 samples/s, utilizing the system’s Conventional Full Body 39-marker model for marker placement. Two force plates (Kistler, Switzerland) recorded bilateral ground reaction forces at a sampling rate of 1,000 samples/s.

The experimental protocol included two rounds, testing two tasks with load weights of 15 kg (low load) and 25 kg (high load) respectively. This is consistent with common lifting studies that use load weight as a variable (Marican et al., 2025). Task order was determined using a balanced randomization procedure. The load was represented by a material box (dimensions: 58 × 40 × 22 cm^3^, empty weight: 10 kg). The load was adjusted by fixing either a 5 kg or 15 kg barbell plate at the center of the box, producing total weights of 15 kg and 25 kg, respectively. Four non-coplanar markers were affixed to the outside of the box for tracking its position.

Upon arrival at the laboratory, the 39 reflective markers were attached to each participant’s anatomical landmarks. The procedure and experimental protocol were explained throughout marker placement. Participants then completed a 10-min warm-up and practiced the lifting motion until fully familiarized with the requirements.

For the formal test, participants stood upright behind the force plates (approximately 20 cm behind), feet shoulder-width apart. When given the start signal, they stepped onto the center of the force plates, stabilized in position, and then performed a deep squat to lift the box, placed in front of the force plates. After lifting, they squatted to place the box back down, returned to standing, and stepped back to the starting position. One cycle was thus completed. Participants were instructed not to move their feet during the lifting and lowering phases. Tasks were repeated until fatigue was reached.

## Fatigue protocol

The fatigue protocol was adapted from prior research and integrated both objective heart rate assessment and the Rating of Perceived Exertion (RPE) using the Borg CR10 scale (Wang et al., 2025).

Heart rate monitoring: A Polar H10 chest-worn heart rate sensor was used to continuously monitor real-time heart rate. Maximum heart rate (MHR) for each participant was estimated using the commonly adopted formula: MHR = 220 − age (in years). Participants performed repeated lifting of the assigned load (15 kg or 25 kg) at a self-paced frequency until their heart rate reached 80% of their estimated MHR.

RPE assessment procedure: Once the 80% MHR threshold was reached, participants were verbally asked to report their RPE score every minute using the Borg CR10 scale (ranging from 0 = “no exertion at all” to 10 = “maximal exertion,” with 18 on the original Borg 6–20 scale corresponding to “very, very hard” on the CR10 scale). The fatigue induction process was terminated when participants reported an RPE score equivalent to 18 on the original scale.

In this study, the average time required to reach fatigue was 5.90 ± 0.72 min for the 15 kg task and 3.94 ± 0.58 min for the 25 kg task. Upon reaching the fatigue threshold, participants immediately performed one final standardized lift to ensure data accuracy for post-fatigue biomechanical analysis.

### Data preprocessing

#### Data filtering

The raw marker coordinate data and three-axis ground reaction force data were processed with a 6 Hz low-pass filter. This filtering step aims to eliminate high-frequency interference signals introduced during measurement, such as those from equipment noise and subjects’ minor non-target movements (e.g., slight body sway). It preserves valid biomechanical signals related to lifting movements, thereby providing a stable and reliable data foundation for subsequent inverse kinematics and inverse dynamics analyses.

#### Data segmentation

In this study, only the data collected while subjects were bearing the external load were analyzed as the lifting movement. The key time points of each trial were determined based on changes in the height of the material box and the sum of ground reaction forces from both force plates:

**Displacement onset of the box:** The z-axis position (vertical direction) of the top marker on the material box was extracted (with x, y, and z representing medial-lateral, anterior-posterior, and superior-inferior axes, respectively) and recorded as z_0_, z_1_, … , z_n_. The difference between successive time points, dᵢ = zᵢ_+1_ - zᵢ, was calculated. The first time point t_1_ where d exceeds 0.1 mm was marked as the displacement onset of the box.

**Movement initiation:** The sum of ground reaction forces from both force plates was calculated from the start of the task up to t_1_, yielding a time series S_0_, S_1_, … , S_t1_. Searching backward from S_t1_, the first local maximum (i.e., the first inflection point of a monotonic increase) was identified as t_0_, and set as the start of the lifting movement.

**Movement termination:** The last time point t_2_ at which d > 0.1 was defined as the end of the movement.

**FIGURE 1:**
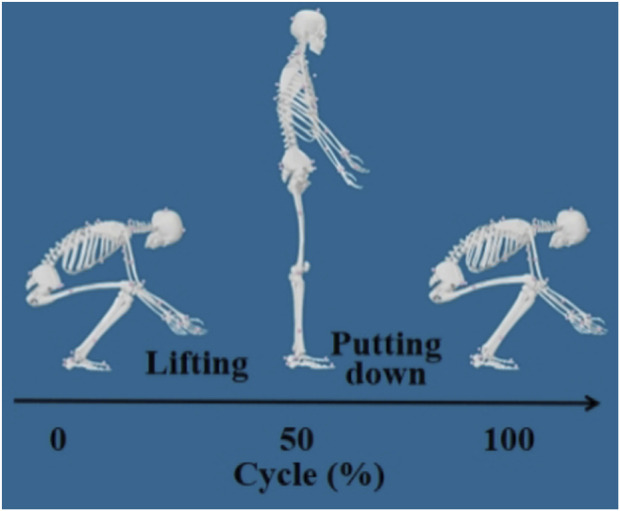
Illustration of the segmented lifting movement.

A schematic of the segmented lifting motion is shown in Figure 1.

#### External force application

Due to experimental limitations, only bilateral ground reaction forces were directly recorded. The forces exerted by the material box on the body were computed and manually applied:

According to Newton’s Second Law (neglecting air resistance), the external forces from the box were: F_x_ = ma_x_, F_y_ = ma_y_, F_z = m·(a_z_ + g), where m is the box mass and g is 9.8 m/s^2^.

As per Newton’s Third Law, the forces exerted by the subject on the box are equal in magnitude and opposite in direction; thus, the external forces applied to the hands were–F_x_, –F_y_, –F_z_.

Kinematic analyses indicated nearly symmetrical limb behavior. Thus, during t_1_ to t_2_, half of each directional force was applied at the location of each hand.

From t_0_ to t_1_ (before the box moves), the horizontal and anterior-posterior external forces on both hands were set to zero. The vertical external force applied at each hand was linearly interpolated from 0 to –½ m g.

### OpenSim analysis

This study utilized OpenSim version 4.4 for analysis, with the complete workflow consisting of four steps. The detailed parameters for each step are as follows:

Data Format Conversion: Using the “C3D File Converter” plugin, raw motion capture data (in C3D format) and force plate data (in TRC format) were converted to. mot and. grf formats compatible with OpenSim, respectively. This ensured temporal synchronization between kinematic and kinetic data.

Model Scaling: Scaling was performed based on the Full-body Musculoskeletal Model of the Lumbar Spine (FBLS), adjusted to match each participant’s anthropometric characteristics (height, weight, and segment lengths calculated from marker positions). The “Scale Model” tool was used for this process, with the following thresholds set: total marker position error <4 cm and root mean square (RMS) error <2 cm. These settings ensured high anatomical matching between the scaled model and each individual participant’s anatomy.

Inverse Kinematics (IK) Analysis: Joint angles (for the hip, knee, and ankle) and other kinematic parameters were calculated using the “Inverse Kinematics” tool. The IK parameter settings were as follows: marker tracking weight = 100, residual tolerance = 1e-5, and smoothing time window = 0.01 s. To reduce data noise, raw kinematic data were filtered using a 6 Hz low-pass Butterworth filter.

Inverse Dynamics (ID) Analysis: With ground reaction forces as input, joint moments, powers, and other kinetic data were obtained using the “Inverse Dynamics” tool. The ID parameter settings were as follows: mass-proportional damping coefficient = 0.05, and a residual elimination algorithm was applied to minimize inconsistencies in kinetic data. For consistency with kinematic data processing, kinetic data were also filtered using a 6 Hz low-pass Butterworth filter.

### Outcome variables and statistical analysis

Outcome variables included both kinematic and kinetic measures.

Kinematic measures: movement duration (s), range of motion (°), average joint angular velocity, and peak joint angular velocity (°/s).

Kinetic measures (normalized to body weight): average joint moment (Nm/kg), peak joint moment (Nm/kg), average joint power (W/kg), peak joint power (W/kg), joint energy expenditure (J/kg).

The joint power and joint energy expenditure indicators were calculated using the joint angles (and angular velocities) obtained from OpenSim inverse kinematics analysis and the joint moments derived from inverse dynamics results. The core formulas are as follows:

(1) Joint Power (p)

Joint power is defined as the product of joint moment and joint angular velocity, reflecting the rate at which work is done by the joint during movement. The formula is:

P=M·ω

M: Net joint moment (in Nm) calculated via inverse dynamics, aligned with the joint’s rotational axis;

*ω*: Joint angular velocity (in rad/s) derived from inverse kinematics, with the same directional reference as the moment (positive when in the same direction, negative when opposite).

(2) Joint Energy Expenditure (E)

Joint energy expenditure is obtained by integrating the absolute value of joint power over the movement duration, representing the total work done by the joint during the movement. The formula is:

$$E=\int_{t_{\text{start}}}^{t_{\text{end}}}\left|P\left(t\right)\right|\text{dt}=\int_{t_{\text{start}}}^{t_{\text{end}}}\left|M\left(t\right)\;\cdot \;\omega \left(t\right)\right|\text{ dt}$$

$t_{\text{start}}$ and $t_{\text{end}}$ : The start and end times of the movement;

$P\left(t\right)$ , $M\left(t\right)$ , $\omega \left(t\right)$ : Time-dependent functions of power, moment, and angular velocity, respectively.

Statistical analysis was conducted using SPSS Statistics v24 (IBM, Armonk, NY, United States). Normality was assessed using the Kolmogorov–Smirnov test. Paired t-tests were used to compare pre- and post-fatigue variables under the same task conditions. The significance level was set at 0.05.

## Results

### Kinematics

#### Movement duration

**FIGURE 2:**
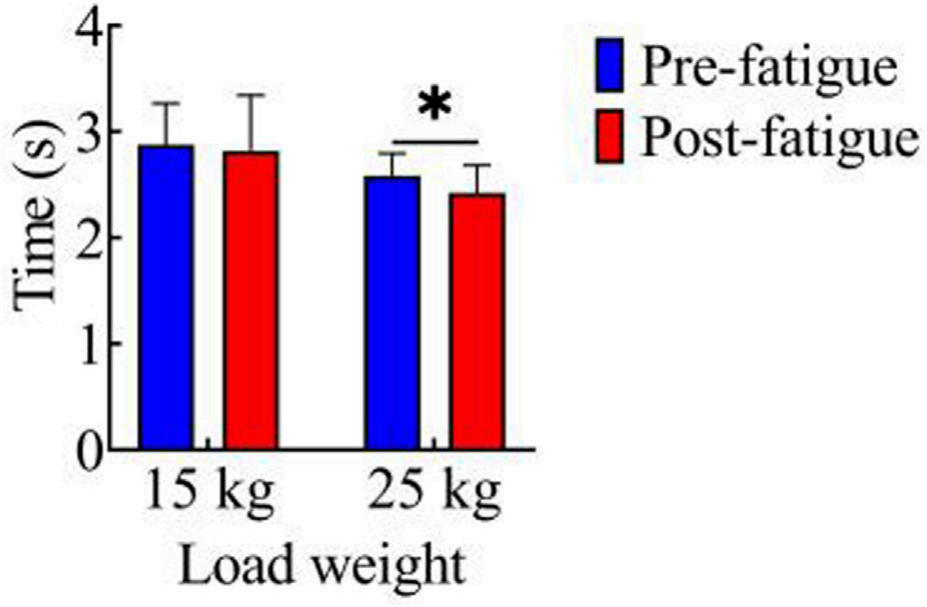
Comparison of movement duration.*, p < 0.05.

Figure 2 shows the comparison of movement duration before and after fatigue in both load conditions. In the 15 kg task, the movement durations before and after fatigue were 2.87 ± 0.39 s and 2.82 ± 0.52 s, respectively, with no significant difference observed between the two (p > 0.05). For the 25 kg task, the movement durations before and after fatigue were 2.59 ± 0.21 s and 2.42 ± 0.25 s, respectively. Notably, the movement duration after fatigue was significantly shorter than that before fatigue (p < 0.05, t = 2.23, Cohen’s d = 0.50, 95% CI = 0.01–0.31).

#### Joint range of motion

**FIGURE 3:**
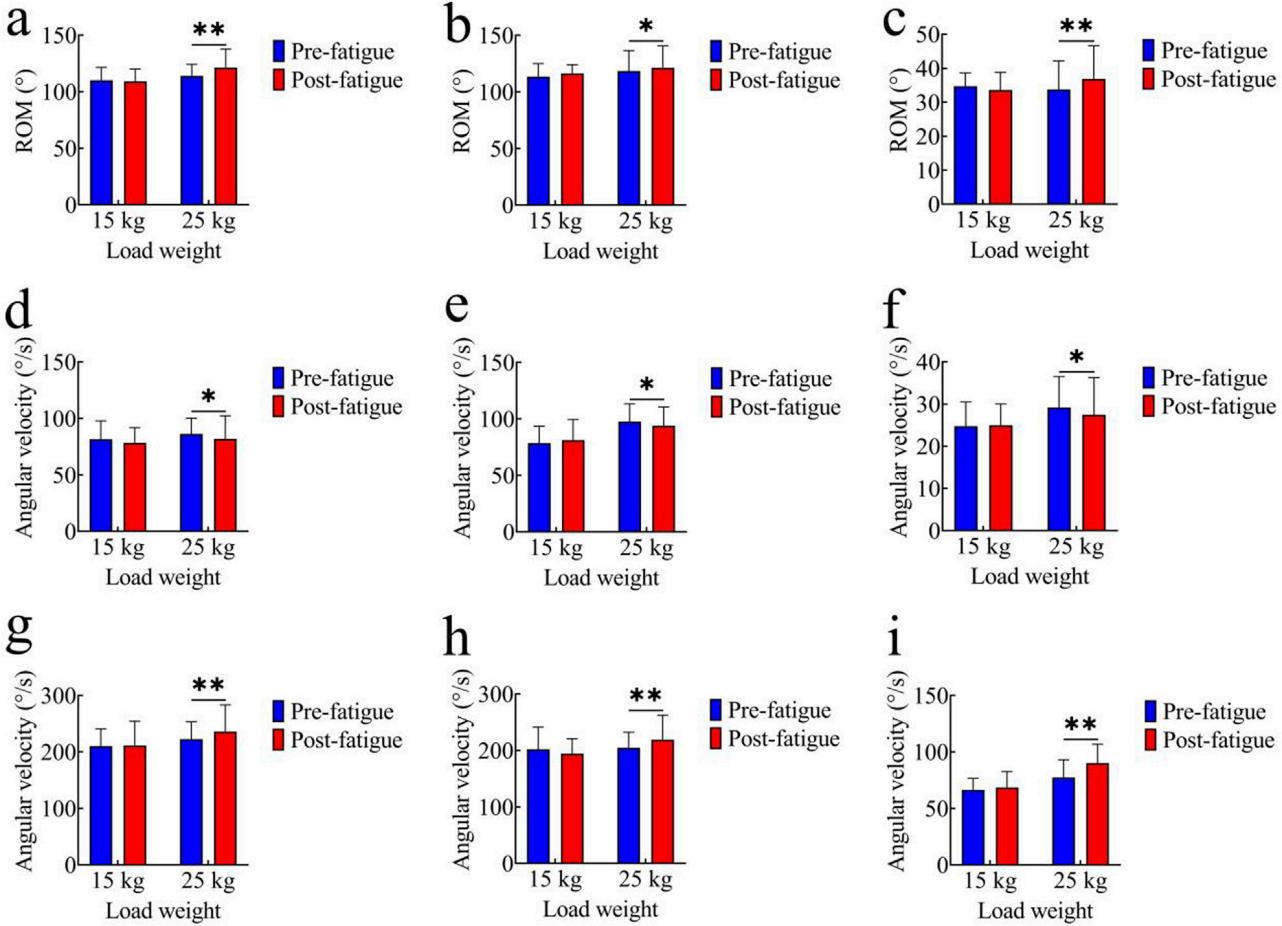
Comparison of kinematic parameters. **(a–c)** Joint range of motion (ROM): **(a)** hip joint; **(b)** knee joint; **(c)** ankle joint; **(d–f)** Average joint angular velocity: **(d)** hip joint; **(e)** knee joint; **(f)** ankle joint; **(g–i)** Peak joint angular velocity:**(g)** hip joint; **(h)** knee joint; **(i)** ankle joint; *, p < 0.05,**, p < 0.01.

Figures 3a–c illustrate the comparison of joint range of motion (ROM) before and after fatigue under both tasks. In the 15 kg task, the pre-fatigue ROM values of each joint were as follows: hip joint: 110.28° ± 11.26°; knee joint: 113.61° ± 11.39°; ankle joint: 34.70° ± 3.94°. After fatigue, the ROM values were: hip joint: 109.21° ± 10.88°; knee joint: 116.42° ± 7.61°; ankle joint: 33.57° ± 10.25°. No significant differences in joint ROM were observed between the pre-fatigue and post-fatigue conditions for the 15 kg task (p > 0.05). In the 25 kg task, the pre-fatigue ROM values of each joint were: hip joint: 113.98° ± 10.30°; knee joint: 118.52° ± 18.01°; ankle joint: 33.72° ± 8.47°. After fatigue, the ROM values were: hip joint: 121.46° ± 16.25°; knee joint: 121.39° ± 19.36°; ankle joint: 36.91° ± 9.72°. Compared with the pre-fatigue condition, the post-fatigue ROM significantly increased for all joints (hip: p < 0.01, t = 4.21, Cohen’s d = 0.94, 95% CI = −11.17 to −3.76; knee: p < 0.05, t = 2.34, Cohen’s d = 0.52, 95% CI = −5.44 to −0.30; ankle: p < 0.01, t = 3.86, Cohen’s d = 0.86, 95% CI = −4.92 to −1.46).

#### Average joint angular velocity

Figures 3d–f compare the average joint angular velocities before and after fatigue. In the 15 kg task, the pre-fatigue average angular velocities of each joint were as follows: hip joint: 81.56° ± 16.22°/s; knee joint: 78.56° ± 15.00°/s; ankle joint: 24.76° ± 5.73°/s. After fatigue, the values were: hip joint: 78.37° ± 13.47°/s; knee joint: 81.16° ± 18.40°/s; ankle joint: 24.99° ± 5.06°/s. No significant differences were observed in the 15 kg task (p > 0.05). In the 25 kg task, the pre-fatigue average angular velocities of each joint were: hip joint: 81.84° ± 20.47°/s; knee joint: 93.93° ± 16.56°/s; ankle joint: 27.47° ± 8.80°/s. After fatigue, the values were: hip joint: 86.31° ± 13.75°/s; knee joint: 97.55° ± 15.81°/s; ankle joint: 29.19° ± 7.33°/s. The average angular velocities of all joints were significantly higher after fatigue (hip: p < 0.05, t = 2.46, Cohen’s d = 0.55, 95% CI = −8.28 to −0.67; knee: p < 0.05, t = 2.59, Cohen’s d = 0.58, 95% CI = −6.55 to −0.70; ankle: p < 0.05, t = 2.59, Cohen’s d = 0.58, 95% CI = −3.12 to −0.22).

#### Peak joint angular velocity

Figures 3g–i show comparisons of peak joint angular velocities. In the 15 kg task, the pre-fatigue peak angular velocities of each joint were as follows: hip joint: 210.41° ± 30.51°/s; knee joint: 202.31° ± 38.96°/s; ankle joint: 66.37° ± 10.23°/s. After fatigue, the values were: hip joint: 211.69° ± 42.64°/s; knee joint: 194.29° ± 26.45°/s; ankle joint: 68.47° ± 14.19°/s. Again, there were no significant differences in the 15 kg task (p > 0.05). In the 25 kg task, the pre-fatigue peak angular velocities of each joint were: hip joint: 222.57° ± 30.63°/s; knee joint: 204.67° ± 27.59°/s; ankle joint: 77.44° ± 15.62°/s. After fatigue, the values were: hip joint: 236.14° ± 47.17°/s; knee joint: 218.99° ± 43.51°/s; ankle joint: 90.05° ± 16.80°/s. The peak angular velocities were significantly higher post-fatigue (hip: p < 0.01, t = 3.11, Cohen’s d = 0.69, 95% CI = −22.71 to −4.43; knee: p < 0.01, t = 3.24, Cohen’s d = 0.72, 95% CI = −23.57 to −5.08; ankle: p < 0.01, t = 2.40, Cohen’s d = 0.54, 95% CI = −23.63 to −1.60).

### Kinetics

#### Average joint torque

**FIGURE 4:**
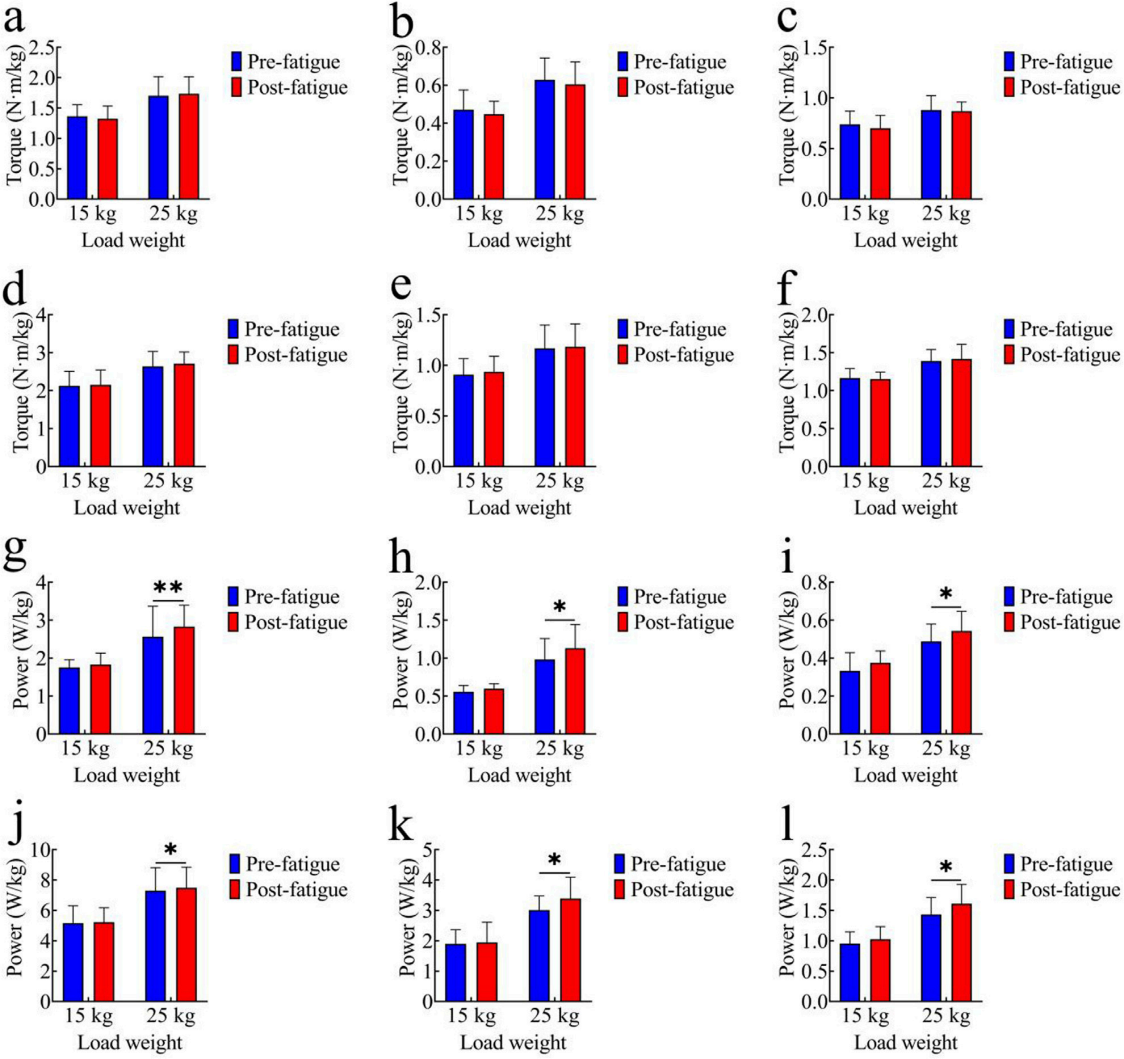
Comparison of kinetic parameters. **(a–c)** Average Joint Torque: **(a)** hip joint; **(b)** knee joint; **(c)** ankle joint; **(d–f)** Peak Joint Torque: **(d)** hip joint; **(e)** knee joint; **(f)** ankle joint; **(g–i)** Average Joint Power: **(g)** hip joint; **(h)** knee joint; **(i)** ankle joint; **(j–l)** Peak Joint Power: **(j)** hip joint; **(k)** knee joint; **(l)** ankle joint; *, p < 0.05,**, p < 0.01.

Figures 4a–c present the comparison of average joint torque before and after fatigue. In the 15 kg task, the pre-fatigue average torque values of each joint were as follows: hip joint: 1.36 ± 0.19 Nm/kg; knee joint: 0.47 ± 0.10 Nm/kg; ankle joint: 0.74 ± 0.13 Nm/kg. After fatigue, the values were: hip joint: 1.32 ± 0.21 Nm/kg; knee joint: 0.45 ± 0.06 Nm/kg; ankle joint: 0.70 ± 0.13 Nm/kg. In the 25 kg task, the pre-fatigue average torque values of each joint were: hip joint: 1.70 ± 0.31 Nm/kg; knee joint: 0.63 ± 0.12 Nm/kg; ankle joint: 0.88 ± 0.14 Nm/kg. After fatigue, the values were: hip joint: 1.73 ± 0.28 Nm/kg; knee joint: 0.60 ± 0.12 Nm/kg; ankle joint: 0.87 ± 0.09 Nm/kg. No significant differences in average joint torque were observed between the pre-fatigue and post-fatigue conditions in either the 15 kg or 25 kg task (p > 0.05).

#### Peak joint torque

Figures 4d–f show peak joint torque data. In the 15 kg task, the pre-fatigue peak torque values of each joint were as follows: hip joint: 2.13 ± 0.38 Nm/kg; knee joint: 0.91 ± 0.16 Nm/kg; ankle joint: 1.16 ± 0.13 Nm/kg. After fatigue, the values were: hip joint: 2.15 ± 0.39 Nm/kg; knee joint: 0.94 ± 0.15 Nm/kg; ankle joint: 1.15 ± 0.09 Nm/kg. In the 25 kg task, the pre-fatigue peak torque values of each joint were: hip joint: 2.64 ± 0.39 Nm/kg; knee joint: 1.17 ± 0.23 Nm/kg; ankle joint: 1.39 ± 0.15 Nm/kg. After fatigue, the values were: hip joint: 2.71 ± 0.30 Nm/kg; knee joint: 1.18 ± 0.23 Nm/kg; ankle joint: 1.42 ± 0.19 Nm/kg. No significant differences in peak joint torque were found between the pre-fatigue and post-fatigue conditions in either the 15 kg or 25 kg task (p > 0.05).

#### Average joint power

Figures 4g–i illustrate average joint power comparisons. In the 15 kg task, the pre-fatigue average power values of each joint were as follows: hip joint: 1.75 ± 0.20 W/kg; knee joint: 0.56 ± 0.08 W/kg; ankle joint: 0.33 ± 0.10 W/kg. After fatigue, the values were: hip joint: 1.83 ± 0.30 W/kg; knee joint: 0.60 ± 0.06 W/kg; ankle joint: 0.38 ± 0.06 W/kg. No significant differences were observed in the 15 kg task (p > 0.05). In the 25 kg task, the pre-fatigue average power values of each joint were: hip joint: 2.57 ± 0.80 W/kg; knee joint: 0.98 ± 0.28 W/kg; ankle joint: 0.49 ± 0.09 W/kg. After fatigue, the values were: hip joint: 2.83 ± 0.57 W/kg; knee joint: 1.13 ± 0.31 W/kg; ankle joint: 0.54 ± 0.10 W/kg. The average joint power was significantly higher after fatigue (hip: p < 0.01, t = 3.91, Cohen’s d = 0.88, 95% CI = −0.40 to −0.12; knee: p < 0.05, t = 2.19, Cohen’s d = 0.49, 95% CI = −0.29 to −0.01; ankle: p < 0.05, t = 2.41, Cohen’s d = 0.54, 95% CI = −0.10 to 0.01).

#### Peak joint power

Figures 4j–l show peak joint power comparisons. In the 15 kg task, the pre-fatigue peak power values of each joint were as follows: hip joint: 5.16 ± 1.14 W/kg; knee joint: 1.89 ± 0.47 W/kg; ankle joint: 0.95 ± 0.19 W/kg. After fatigue, the values were: hip joint: 5.22 ± 0.95 W/kg; knee joint: 1.94 ± 0.67 W/kg; ankle joint: 1.02 ± 0.21 W/kg. No significant differences were observed in the 15 kg task (p > 0.05). In the 25 kg task, the pre-fatigue peak power values of each joint were: hip joint: 7.30 ± 1.51 W/kg; knee joint: 3.01 ± 0.47 W/kg; ankle joint: 1.43 ± 0.28 W/kg. After fatigue, the values were: hip joint: 7.49 ± 1.36 W/kg; knee joint: 3.39 ± 0.70 W/kg; ankle joint: 1.61 ± 0.32 W/kg. The peak joint power was significantly higher post-fatigue (hip: p < 0.05, t = 2.13, Cohen’s d = 0.48, 95% CI = −0.37 to −0.01; knee: p < 0.05, t = 2.53, Cohen’s d = 0.56, 95% CI = −0.70 to −0.07; ankle: p < 0.05, t = 2.15, Cohen’s d = 0.48, 95% CI = −0.35 to −0.01).

#### Energy expenditure

**FIGURE 5:**
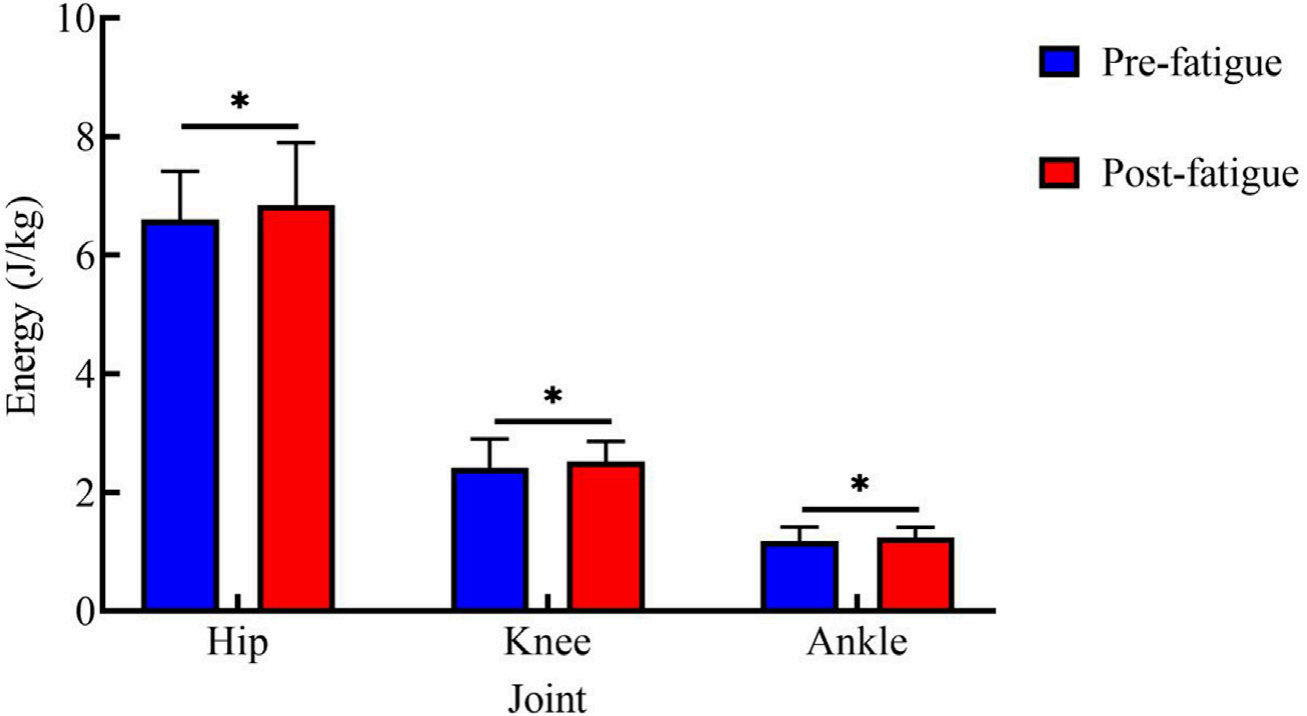
Energy Expenditure Comparison in 25 kg Tasks. *, p < 0.05.

Figure 5 shows energy expenditure at each joint during the 25 kg task. Before fatigue, the energy expenditure values of each joint were as follows: hip joint: 6.60 ± 0.81 J/kg; knee joint: 2.41 ± 0.49 J/kg; ankle joint: 1.18 ± 0.24 J/kg. After fatigue, the values were: hip joint: 6.84 ± 1.05 J/kg; knee joint: 2.52 ± 0.34 J/kg; ankle joint: 1.24 ± 0.17 J/kg. After fatigue, the energy expenditure of all joints was significantly higher than that before fatigue (hip: p < 0.05, t = 2.74, Cohen’s d = 0.61, 95% CI = −0.43 to −0.06; knee: p < 0.05, t = 2.50, Cohen’s d = 0.56, 95% CI = −0.20 to −0.02; ankle: p < 0.01, t = 3.40, Cohen’s d = 0.76, 95% CI = −0.10 to −0.02).

## Discussion

This study analyzed the kinematic and kinetic characteristics of healthy young men performing lifting tasks of 15 kg and 25 kg before and after fatigue, to explore how subjects adapt and adjust their movement strategies in response to accumulated fatigue. Through inverse dynamics analysis with OpenSim, the study further examined the negative impacts of fatigue-induced changes in kinematic parameters from a joint kinetics perspective.

The key findings of this study are as follows: (1) Under the 15 kg low-load condition, neither kinematic parameters (movement duration, joint range of motion, average/peak joint angular velocity) nor kinetic parameters (joint torque, power, energy expenditure) of the hip, knee, and ankle showed significant differences before and after fatigue; (2) Under the 25 kg high-load condition, fatigue led to significant increases in joint range of motion, average/peak joint angular velocity, and movement rhythm acceleration in kinematic indicators, while in kinetic indicators, average/peak joint torque remained stable, but average power, peak power, and energy expenditure increased significantly. The following discussion is based on these core results to explore the underlying mechanisms and implications.

### Effects of fatigue on kinematic characteristics

In this experiment, participants performed lifting tasks with 15 kg and 25 kg loads, and their lower limb kinematic characteristics were quantified before and after fatigue. The results showed that under the 15 kg load, there were no significant differences in movement duration, joint range of motion, or average and peak joint angular velocity of the hip, knee, and ankle, regardless of fatigue state. In contrast, under the 25 kg load, a clear acceleration in movement rhythm was observed after fatigue, along with significant increases in joint range of motion and angular velocity parameters across all three joints. These findings reveal the adaptive regulation and changes in human motor control in response to different load and fatigue conditions.

The stability of lower limb kinematics under low-load (15 kg) conditions can be attributed to the efficient self-organization and energy allocation of the neuromuscular system. Previous research has indicated that in repetitive or sustained low-intensity physical labor, although some muscle groups may experience fatigue, overall motor performance remains stable. The body compensates locally and through movement proficiency, maintaining consistent posture and movement trajectories (Lu et al., 2015). This phenomenon has also been documented in studies of balance and gait recovery, where the variability of spatiotemporal parameters such as joint angles and velocities remains low under low-load or mild fatigue conditions (Gates and Dingwell, 2011).

However, as the load increases, the impact of fatigue on movement control becomes more prominent. Under a high load (25 kg), fatigue prompted subjects to use faster movement speeds and increase both joint range of motion and angular velocity in order to complete the task. On one hand, this may represent the body’s response to reduced output from core muscle groups, an attempt to accelerate task completion and mitigate discomfort from sustained high-intensity exertion—i.e., shortening the duration of single movements to redistribute the sensation of fatigue (Gates and Dingwell, 2011). On the other hand, increases in joint excursion and speed reflect greater reliance on extreme joint movements under high-load, high-fatigue conditions to successfully execute lifting (Saraceni et al., 2021).

Repeated high-load tasks can lead to altered lower limb movement trajectories and joint control strategies. Previous studies have found that muscle fatigue significantly affects movement coordination and kinematic variability, especially under heavy loads (Madigan and Pidcoe, 2003). Furthermore, *in situ* fatigue experiments revealed that fatigue changes lower limb stiffness and movement strategies, further affecting joint mobility and spatiotemporal characteristics; sometimes, larger and faster joint movements are used as compensatory mechanisms to maintain movement efficiency (Padua et al., 2006).

While such adaptations may support task completion in the short term, they also pose health risks. After fatigue, both joint range of motion and angular velocity increase, while motor control capability declines. These changes may potentially increase the rates of soft tissue strains and joint sprains; if uncorrected over time, they might further exacerbate chronic musculoskeletal burdens (Xi et al., 2025). It should be noted that the participants in this study were healthy students, and no direct injury outcomes were measured. This inference about potential risk requires verification by subsequent longitudinal studies or occupational population investigations. Studies have shown that lifting and carrying under fatigue decreases lumbar and lower limb coordination and increases movement amplitude—a pattern closely linked to work-related lower limb injuries in occupational populations, suggesting similar potential risks in our fatigue-induced kinematic changes (Xi et al., 2025).

Fatigue is influenced by multiple variables, including load weight, task duration, and individual fitness. Greater load and longer task duration accelerate fatigue accumulation and lead to more pronounced movement changes. Once a high-intensity task reaches the fatigue threshold, self-adjustment of posture and movement speed becomes aggressive, imposing additional stress on the musculoskeletal system (Yi et al., 2022). Moreover, factors such as sex, age, and work experience also moderate the extent of post-fatigue changes in movement strategy (K et al., 2023).

Finally, fatigue disrupts the integration of postural and kinetic information, resulting in delayed control and fragmented movements during complex lifting tasks. Some subjects, under high-load fatigue, unconsciously adopt higher and faster joint swings to compensate for strength loss, or employ joint locking as a protective adjustment. Such changes decrease movement redundancy and coordination stability, which are crucial mechanisms underlying increased lower limb injury risk—key mechanisms that may contribute to increased lower limb injury risk, though this needs confirmation in studies with injury outcome measures (Antonopoulos et al., 2014).

## Adverse effects of changes in kinematic characteristics

Kinetic results showed that when lifting a 15 kg load, there were no significant differences in the kinetic parameters of the hip, knee, and ankle joints before and after fatigue. In contrast, during the 25 kg high-load lifting task, while average and peak joint torques did not show statistically significant changes, average power, peak power, and energy expenditure at the hip, knee, and ankle joints all increased significantly with accumulated fatigue. These findings reveal the profound impact of the combined effects of muscle fatigue and high loading on lower limb kinetic regulation, highlighting the deteriorating trend of mechanical efficiency and potential health risk under fatigue.

First, under the 15 kg loading condition, none of the major kinetic indicators changed significantly regardless of fatigue status, which is consistent with previous literature. Brief periods of low-to moderate-intensity lifting rarely push the joints or primary muscle groups to their mechanical limits; thus, the neuromuscular system can flexibly allocate torque and power through mechanisms such as motor redundancy and muscle synergy, ensuring economical and stable movements (Bonato et al., 2003). For instance, experimental evidence shows that, during repetitive low- or moderate-intensity lifting tasks, peak joint torques and average joint power remain largely stable as long as the fatigue threshold has not been crossed. By recruiting more motor units and adjusting muscle activation sequences, the motor control system can compensate for initial decreases in local muscle output, producing a “stable plateau” in overall kinetic output (Enoka and Duchateau, 2008). Therefore, the lack of significant changes in torque, power, and energy expenditure of the 15 kg group in this study can be regarded as a normal physiological regulation.

Under the 25 kg high-load condition, the distinct changes in kinetic parameters (stable torque but increased power and energy expenditure) reflect the specific adaptation of the neuromuscular system to the combined stress of high load and fatigue. Torque stability under high-load fatigue may be related to the body’s maintenance of basic force output requirements—since torque directly determines the ability to overcome external loads, the neuromuscular system prioritizes stabilizing torque to ensure task completion (Kirsch and Rymer, 1987). This is consistent with the finding that dynamic torque tends to be maintained in the early stages of fatigue, while power changes are more sensitive to fatigue progression (Swisher et al., 2019). In contrast, power and energy expenditure showed significant increases, which are closely linked to the combined effects of torque maintenance and kinematic changes, as well as intrinsic neuromuscular inefficiencies (Doke and Kuo, 2007).

The core mechanical relationship explaining the “stable torque but increased power” phenomenon lies in the definition of mechanical power: power = torque × angular velocity. In this study, under high-load fatigue, joint torque remained stable, but joint angular velocity increased significantly (as shown in the kinematic results), and the product of the two—joint power—thus increased accordingly. This mechanical coupling effect is further amplified by neuromuscular changes. On one hand, fatigue-induced reduction in muscle synergy efficiency leads to the need for recruiting more motor units, especially fast-twitch muscle fibers (Chumanov et al., 2007); fast-twitch fibers have lower mechanical and metabolic efficiency, requiring more energy input to maintain torque output and supporting higher angular velocities, thereby increasing power and energy expenditure. On the other hand, fatigue impairs movement coordination, resulting in scattered and disordered movement patterns (Cowley and Gates, 2017); this increases “ineffective work” during the task, as the body needs to consume additional energy to adjust movement deviations, which further elevates energy expenditure even when torque is stable (Gagnat et al., 2023). A previous study on dynamic fatigue also report that, after fatigue, lifting movements become more fragmented and the paths of force flow are altered, indirectly increasing total energy expenditure, which aligns with our observation that increased angular velocity drives power elevation while torque remains stable (Madigan and Pidcoe, 2003).

Additionally, higher average and peak joint power indicate that joints are reaching higher peaks in force output and energy consumption within short timeframes. In occupational settings with long-term repetitive lifting, increased peak power has been reported to enhance the capacity for short-duration tasks but also increases the mechanical burden on soft tissues and joint structures, which may contribute to potential acute injuries such as strains and sprains (Dolan and Adams, 1998). Frequent exposure to high power peaks has also been identified as a potential mechanism for chronic injuries and pain syndromes in long-term manual workers (Montero and Lundby, 2015). Longitudinal studies among manual workers reveal that for every 10% increase in power or energy expenditure, the rate of related injuries and long-term functional decline rises proportionally (Viljoen et al., 2021). However, it should be emphasized that the participants in this study were healthy university students without measured injury indicators, so these inferences mainly point to potential risk mechanisms in long-term occupational contexts rather than confirmed injury outcomes in the current sample.

It is important to note that, while changes in torque were not observed here, the increase in power and energy consumption essentially reflects a “high-cost” compensatory strategy for fatigue. Some reports suggest that, under extreme fatigue or with very frequent high-load work, joint torques may actually decrease, movement amplitude may decline, and eventually efficiency breaks down completely (the so-called “kinetic trough” phenomenon) (Potvin and Fuglevand, 2017). However, during early and intermediate fatigue stages, the body typically prioritizes high-energy systems to maintain force output, which explains the concurrent rise in power and energy expenditure observed in this study (Miller et al., 1993).

Combining insights from biomechanics and neuromuscular science, reduced force production efficiency, weakened motor synergies, protective relative immobility, and fragmented movement have all been established as hallmarks of how fatigue drives adverse kinetic adaptations in lifting tasks (Granata and Wilson, 2001). More specifically, the hip, knee, and ankle joints fulfill different roles: the hip coordinates core force transfer and large postural adjustments; the knee is responsible for primary power generation and shock absorption; and the ankle manages ground reaction conversion and adaptation (Anderson and Blake, 1994). Under high-load fatigue, all three joints must work in coordination, and the observed simultaneous increases in joint power and energy further underscore the high energy demands and potential risk burdens of such compensatory adaptations in long-term work scenarios.

## Limitations

This study has the following limitations: First, the sample is homogeneous, including only healthy young male college students, without actual manual laborers or individuals of different ages and genders, which limits the generalizability of the study conclusions. Second, fatigue was determined solely based on heart rate (reaching 80% of maximum heart rate) and the Rating of Perceived Exertion (RPE = 18), without using more direct indicators for assessing local muscle fatigue. Third, the load setting only included two levels (15 kg and 25 kg), failing to cover a broader range of load gradients. Finally, the study design only focused on the acute effects of fatigue on lower limb biomechanics, without involving long-term impacts, and did not incorporate electromyography (EMG)-related indicators for auxiliary analysis.

## Conclusion

Under the 15 kg lifting condition, primary kinematic and kinetic indicators of lower limb joints remained stable before and after fatigue, suggesting strong resistance to fatigue. In contrast, for 25 kg high-load lifting, fatigue drove faster movement rhythms and significantly increased joint range of motion, angular velocity, power, and energy expenditure. The combined effect of high load and fatigue not only increased the burden on the musculoskeletal system but also led to a rise in potential injury risk.

However, the participants in this study were limited to healthy male college students, and the sample was not extended to actual manual laborers or populations of different ages and genders. Therefore, further verification is required to determine whether these conclusions are applicable to all populations. Nevertheless, the results of this study can provide a reference for occupational health management, such as the reasonable regulation of material lifting intensity, ensuring fatigue recovery for workers, and alleviating the lower limb burden of workers.

## Data Availability

The raw data supporting the conclusions of this article will be made available by the authors, without undue reservation.
